# Supplementary material for: Thermogenic Activation Downregulates High Mitophagy Rate in Human Masked and Mature Beige Adipocytes
Source: Int J Mol Sci. 2020 Sep 10;21(18):6640. doi: 10.3390/ijms21186640 (PMC7555361; doi:10.3390/ijms21186640)
Supplement: Supplementary file 1 [file ijms-21-06640-s001.zip › Supplementary materials.pdf]

SUPPLEMENTARY MATERIALS

Thermogenic activation downregulates high mitophagy rate in human masked and mature beige adipocytes

Mária Szatmári-Tóth, Abhirup Shaw*, István Csomós, Gábor Mocsár, Pamela Fischer-Posovszky, Martin Wabitsch, Zoltán Balajthy, Cecília Lányi, Ferenc Győry, Endre Kristóf, László Fésüs***

* Contributed equally

**Corresponding author:

László Fésüs, MD PhD DSc MHASc
Department of Biochemistry and Molecular Biology
Faculty of Medicine, University of Debrecen
H-4032, Debrecen, Egyetem tér 1., Hungary
Tel: +3652-416-432, Fax: +3652-314-989
E-mail address: fesus@med.unideb.hu

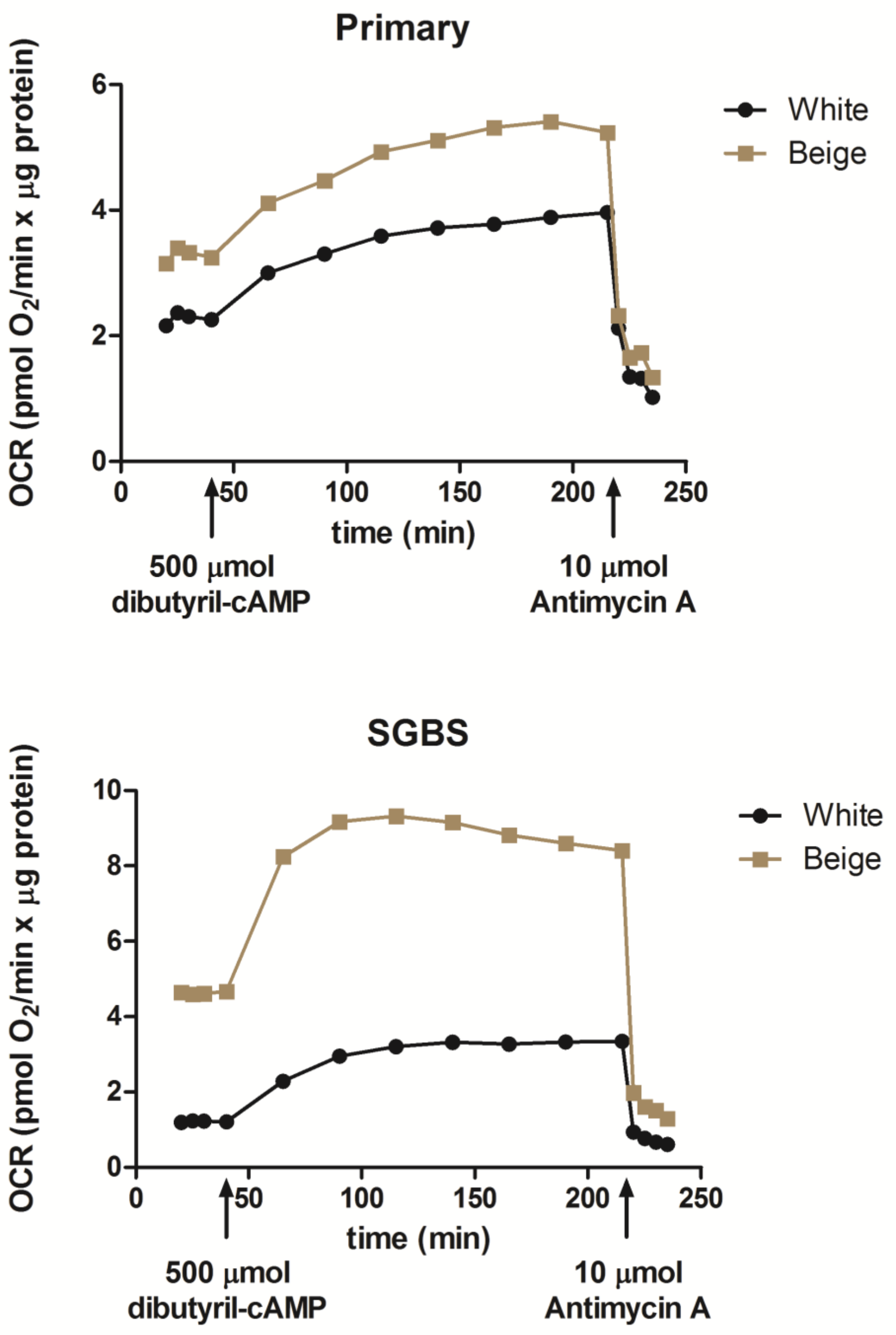

Supplementary figure 1. Oxygen consumption of one representative hASC-derived adipocyte donor and SGBS adipocyte sample measured by XF96 oximeter as described earlier [29,30]. After recording the baseline oxygen consumption, cells received a single bolus dose of dibutyril-cAMP (500 µM final concentration) modelling adrenergic stimulation. Then, stimulated oxygen consumption was recorded every 30 min. The oxygen consumption rate was normalized to protein content and normalized readings were displayed.

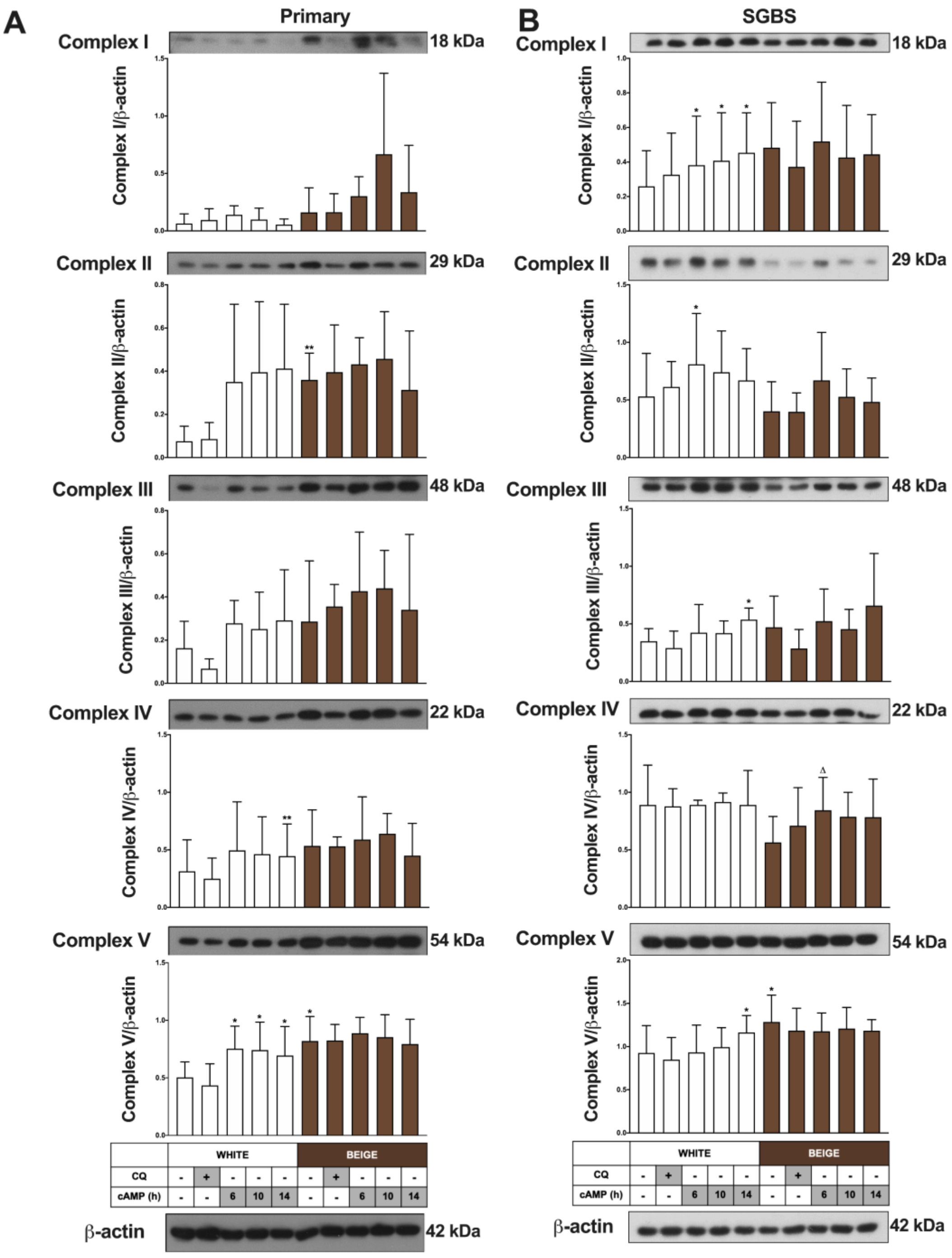

Supplementary figure 2. Expression of mitochondrial complex subunits in adipocytes upon cAMP treatment. Adipocytes were differentiated and treated as in Figure 1. Representative immunoblots and densitometry analysis of mitochondrial complex subunits protein expression (A) in hASC-derived (n=4) and (B) in SGBS cells (n=5) normalized to β-actin. Results are expressed as mean±SD. Statistics: two-tailed paired student t-test, *$p<0.05$, **$p<0.01$, *: significant as compared to white untreated sample, Δ$p<0.05$, ΔΔ$p<0.01$, Δ: significant as compared to beige untreated sample.

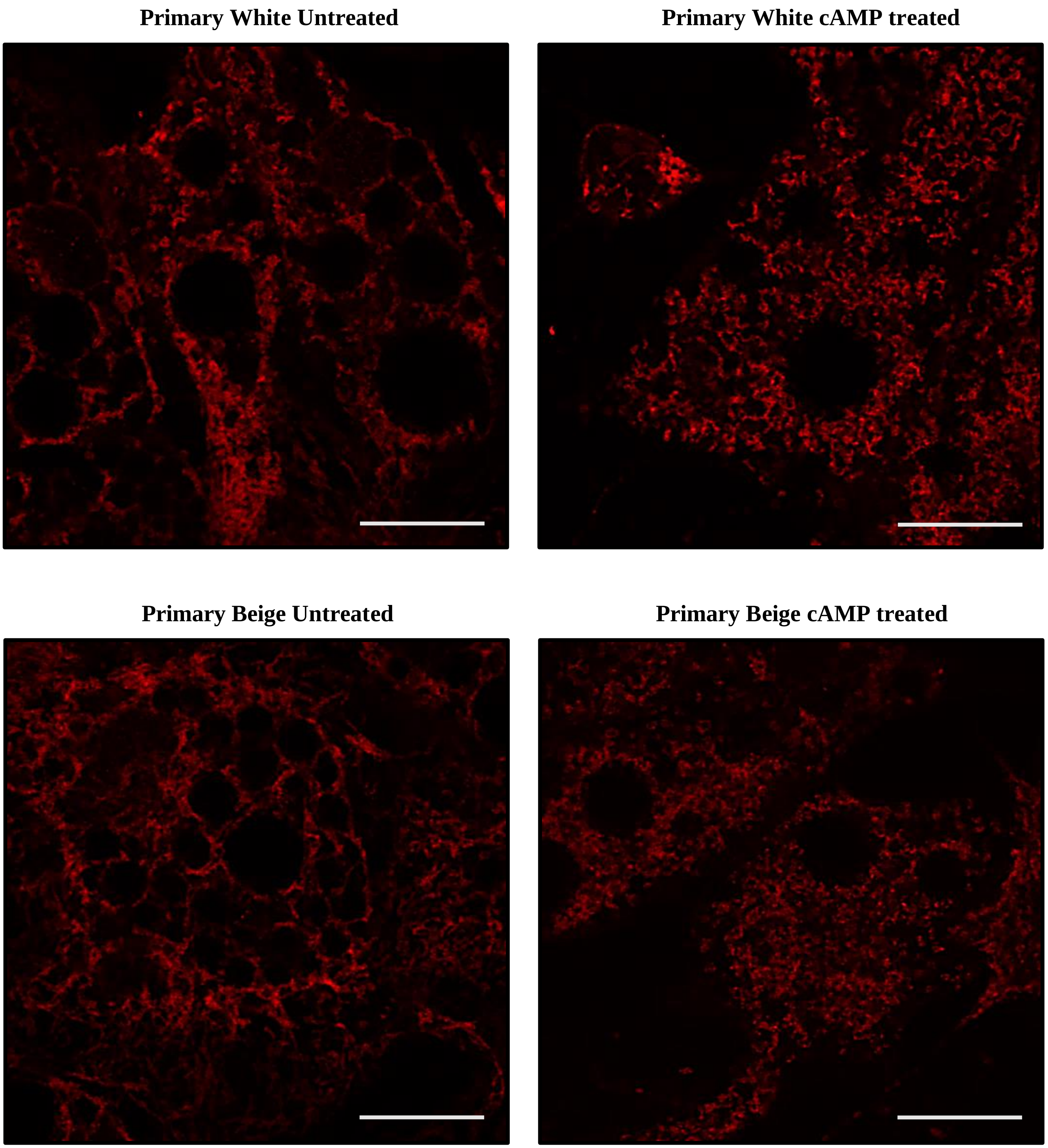

Supplementary figure 3. High-resolution confocal microscopy images of TOM20 immunostaining. Scale bars represent 5 µm.

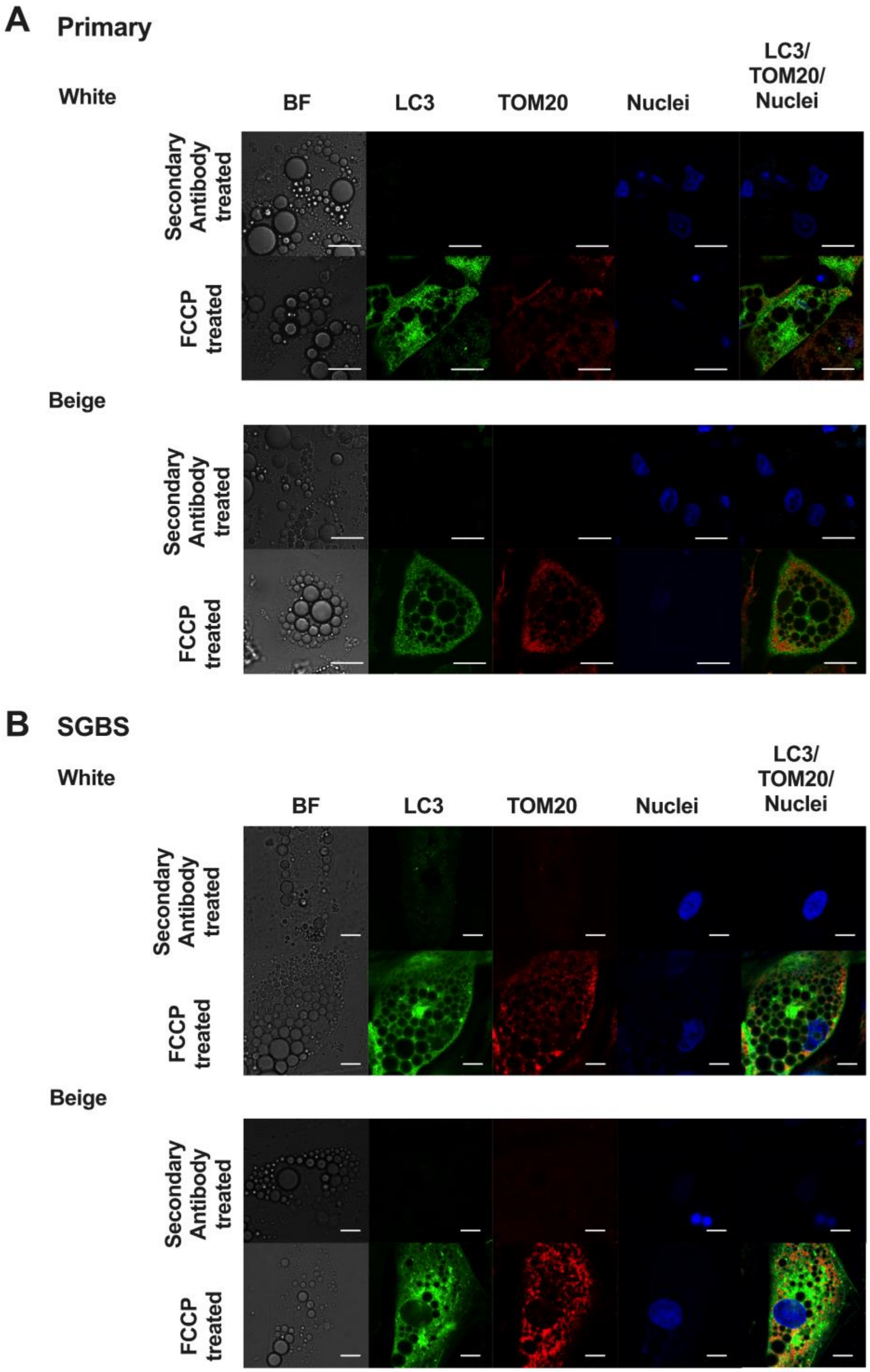

Supplementary figure 4. Confocal images of LC3 and TOM20 immunostaining in control and FCCP treated samples. Representative confocal microscopy images of secondary antibody controls for LC3 and TOM20 immunostaining in white (up) and beige (down) adipocytes and upon FCCP treatment (10 µM, 6 h) (A) in primary and (B) in SGBS cells.

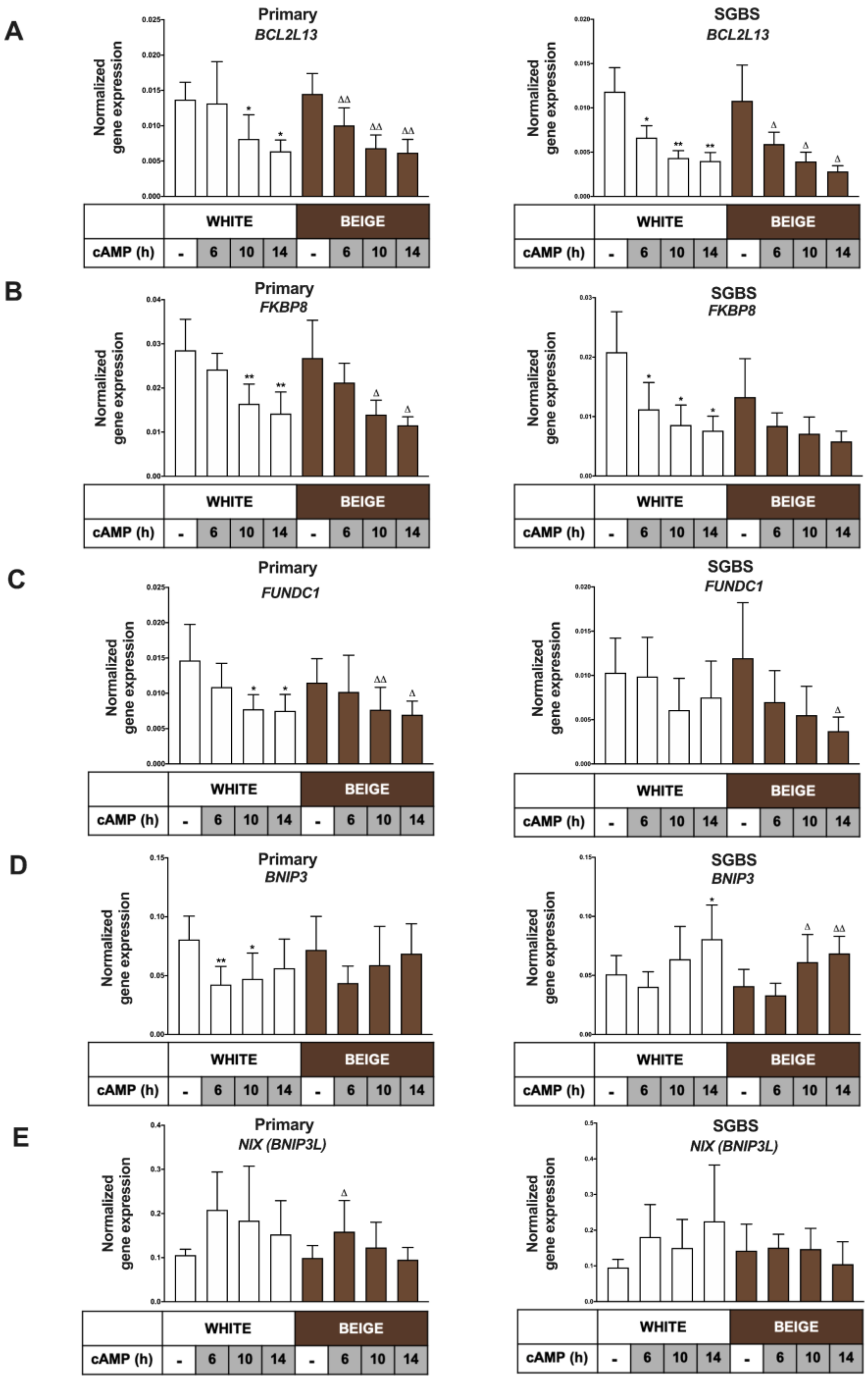

Supplementary figure 5. Expression of genes involved in Parkin-independent mitophagy is decreased as a result of cAMP stimulus. Adipocytes were differentiated and treated as in Figures 1 and 2. Gene expression of (A) BCL2L13, (B) FKBP8, (C) FUNDC1, (D) BNIP3, (E) NIX (BNIP3L) in hASC-derived (left) and SGBS cells (right) (n=5). Gene expression was determined by RT-qPCR and target genes were normalized to GAPDH. Results are expressed as mean±SD. Statistics: two-tailed paired student t-test, *$p<0.05$, **$p<0.01$, *: significant as compared to white untreated sample, **Δ**$p<0.05$, **ΔΔ**$p<0.01$, **Δ:** significant as compared to beige untreated sample.
