# Supplementary material for: Thermogenic Activation Downregulates High Mitophagy Rate in Human Masked and Mature Beige Adipocytes
Source: Int J Mol Sci. 2020 Sep 10;21(18):6640. doi: 10.3390/ijms21186640 (PMC7555361; doi:10.3390/ijms21186640)
Supplement: Supplementary file 1 [file ijms-21-06640-s001.zip › All original western blots.pdf]

Original western blots for Figure 1 B

Primary

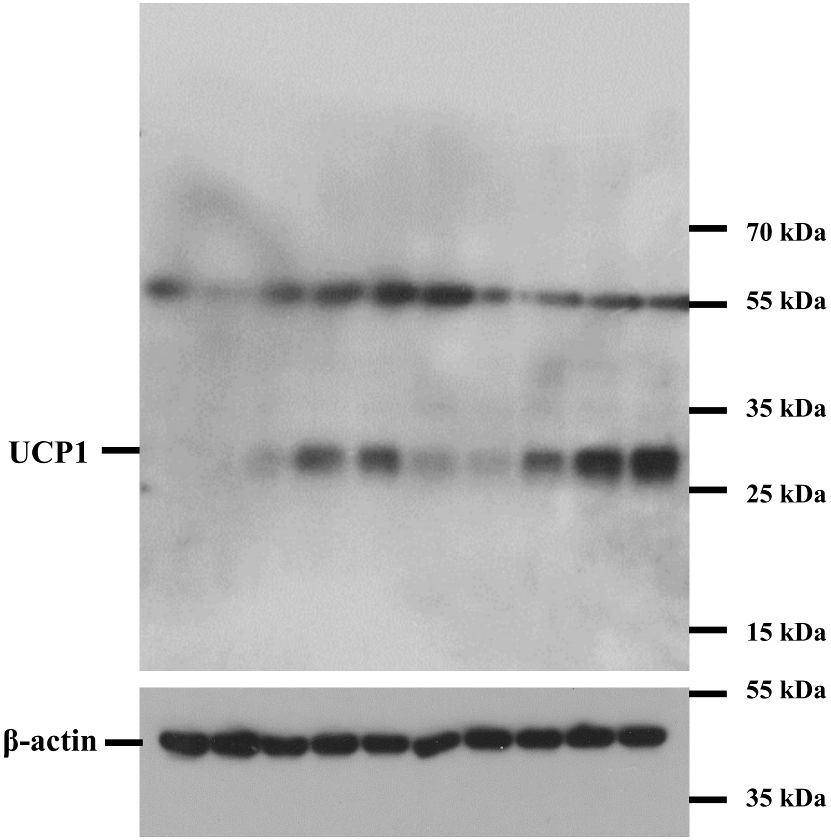

	WHITE					BEIGE				
CQ	-	+	-	-	-	-	+	-	-	-
cAMP (h)	-	-	6	10	14	-	-	6	10	14

SGBS

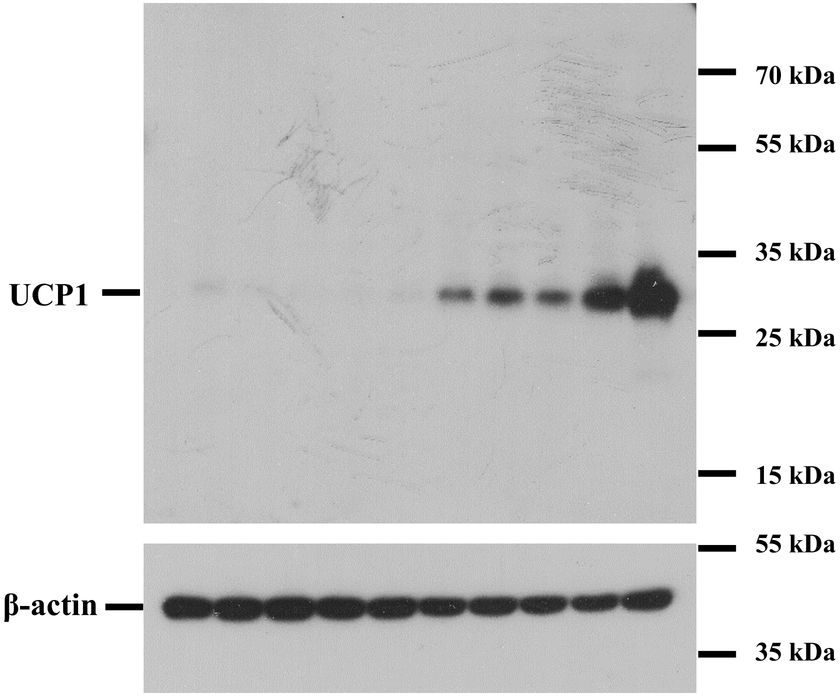

	WHITE					BEIGE				
CQ	-	+	-	-	-	-	+	-	-	-
cAMP (h)	-	-	6	10	14	-	-	6	10	14

Original western blots for Figure 2 C

Primary

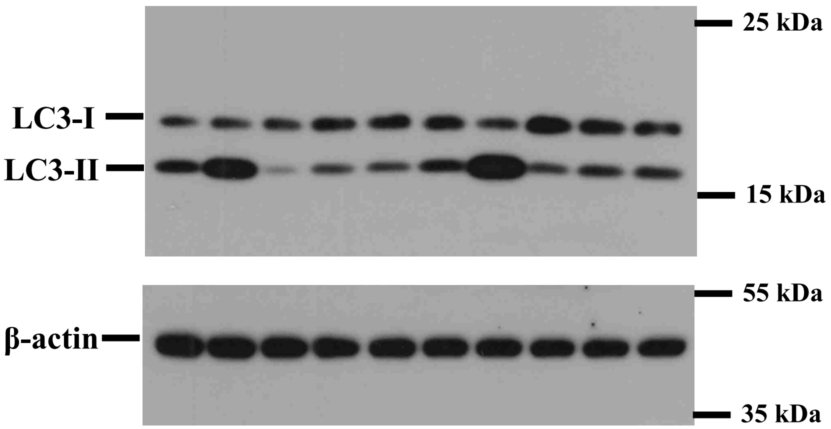

	WHITE					BEIGE				
CQ	-	+	-	-	-	-	+	-	-	-
cAMP (h)	-	-	6	10	14	-	-	6	10	14

Primary

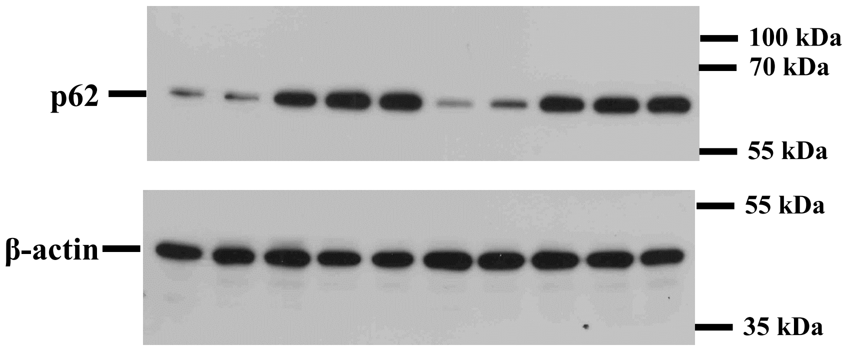

	WHITE					BEIGE				
CQ	-	+	-	-	-	-	+	-	-	-
cAMP (h)	-	-	6	10	14	-	-	6	10	14

Original western blots for Figure 2 D

SGBS

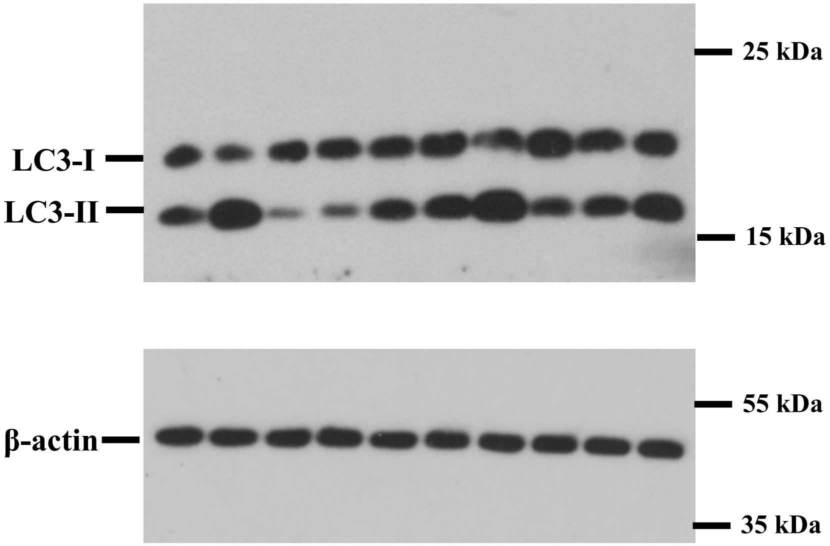

	WHITE					BEIGE				
CQ	-	+	-	-	-	-	+	-	-	-
cAMP (h)	-	-	6	10	14	-	-	6	10	14

SGBS

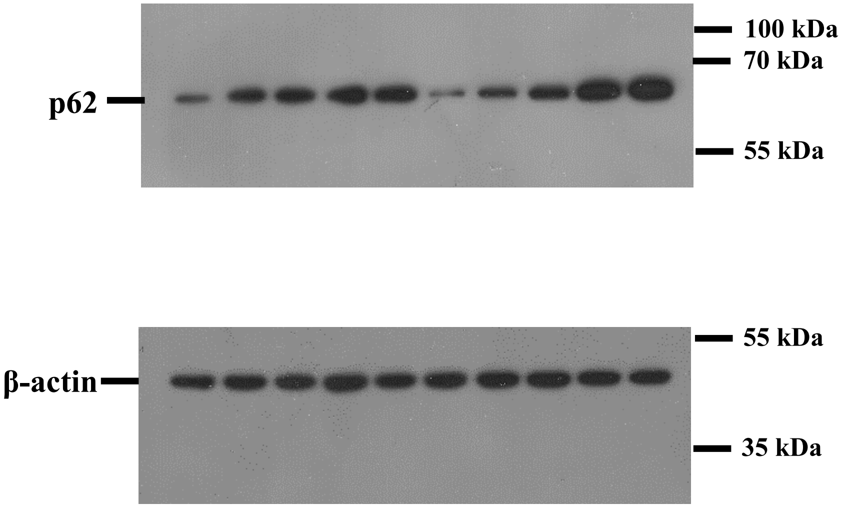

	WHITE					BEIGE				
CQ	-	+	-	-	-	-	+	-	-	-
cAMP (h)	-	-	6	10	14	-	-	6	10	14

Original western blots for Figure 4 A

Primary

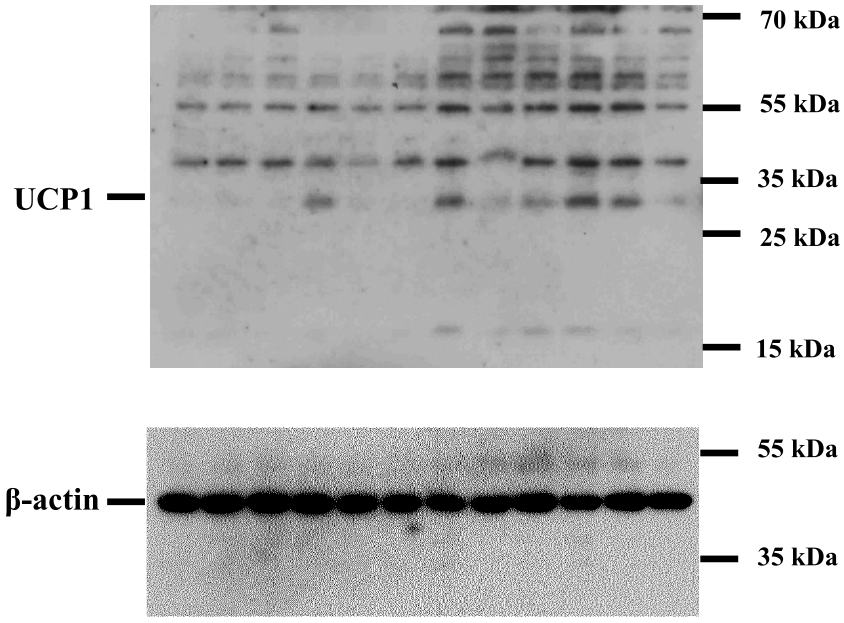

	WHITE						BEIGE					
CQ	-	+	-	-	+	-	-	+	-	-	+	-
H89	-	-	+	-	-	+	-	-	+	-	-	+
cAMP	-	-	-	+	+	+	-	-	-	+	+	+

SGBS

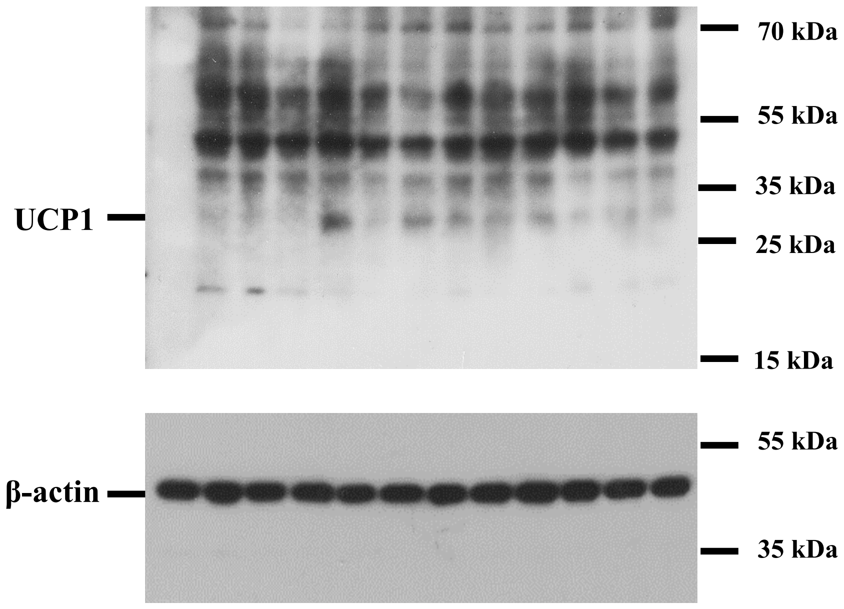

	WHITE						BEIGE					
CQ	-	+	-	-	+	-	-	+	-	-	+	-
H89	-	-	+	-	-	+	-	-	+	-	-	+
cAMP	-	-	-	+	+	+	-	-	-	+	+	+

Original western blots for Figure 4 B

Primary

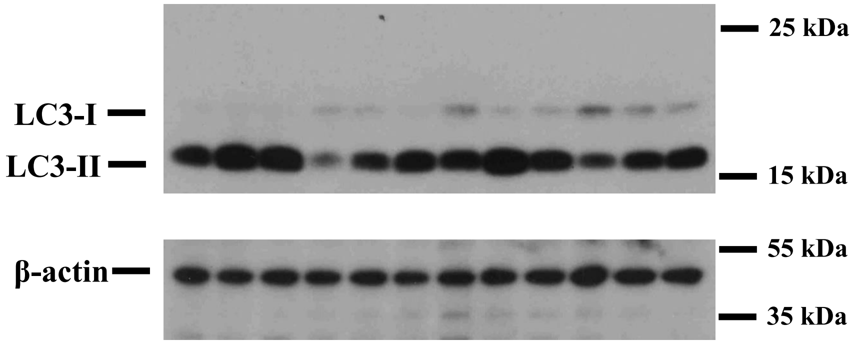

	WHITE						BEIGE					
CQ	-	+	-	-	+	-	-	+	-	-	+	-
H89	-	-	+	-	-	+	-	-	+	-	-	+
cAMP	-	-	-	+	+	+	-	-	-	+	+	+

SGBS

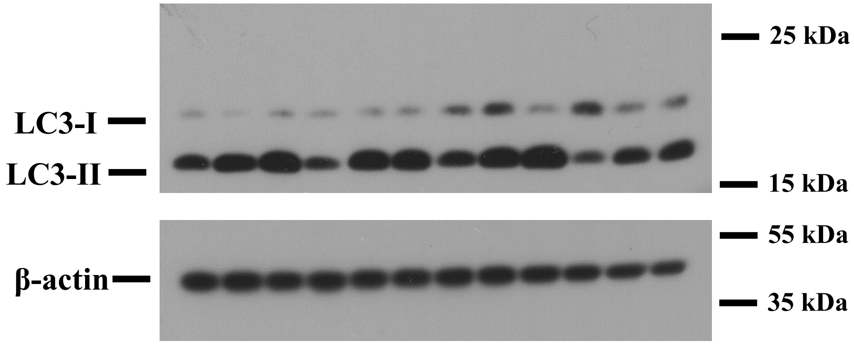

	WHITE						BEIGE					
CQ	-	+	-	-	+	-	-	+	-	-	+	-
H89	-	-	+	-	-	+	-	-	+	-	-	+
cAMP	-	-	-	+	+	+	-	-	-	+	+	+

Original western blots for Figure 4 C

Primary

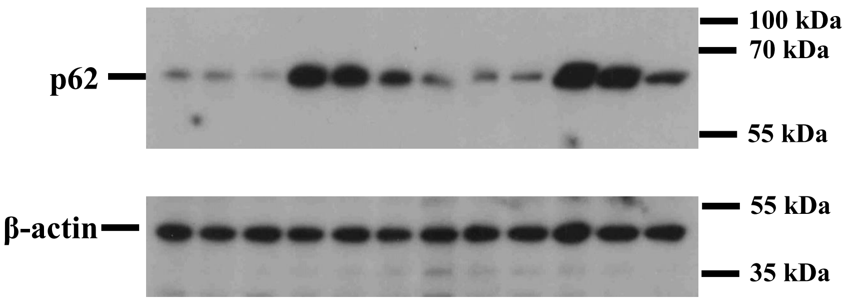

	WHITE						BEIGE					
CQ	-	+	-	-	+	-	-	+	-	-	+	-
H89	-	-	+	-	-	+	-	-	+	-	-	+
cAMP	-	-	-	+	+	+	-	-	-	+	+	+

SGBS

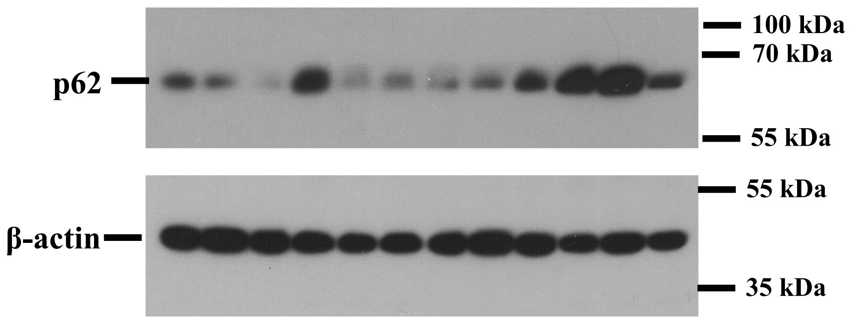

	WHITE						BEIGE					
CQ	-	+	-	-	+	-	-	+	-	-	+	-
H89	-	-	+	-	-	+	-	-	+	-	-	+
cAMP	-	-	-	+	+	+	-	-	-	+	+	+

Original western blots for Figure 5 B

Primary

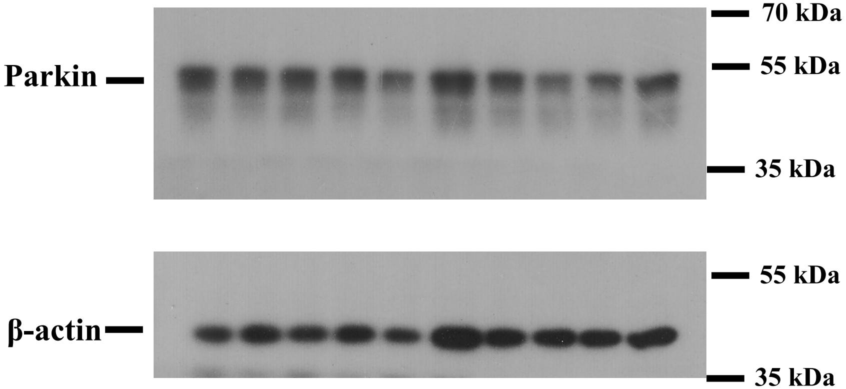

	WHITE					BEIGE				
CQ	-	+	-	-	-	-	+	-	-	-
cAMP (h)	-	-	6	10	14	-	-	6	10	14

SGBS

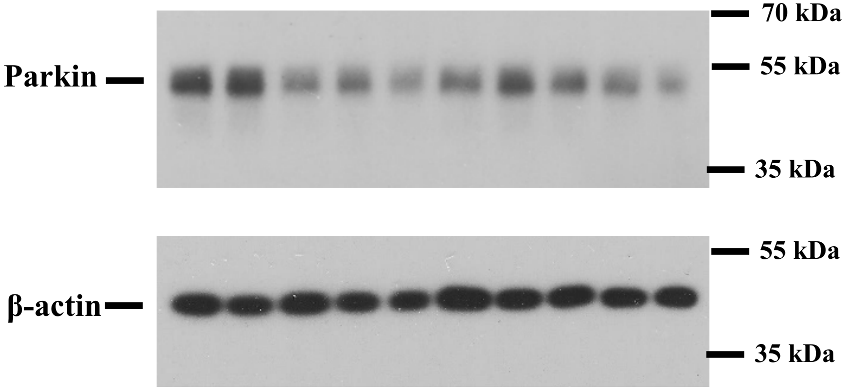

	WHITE					BEIGE				
CQ	-	+	-	-	-	-	+	-	-	-
cAMP (h)	-	-	6	10	14	-	-	6	10	14

Original western blots for Figure 6

Primary

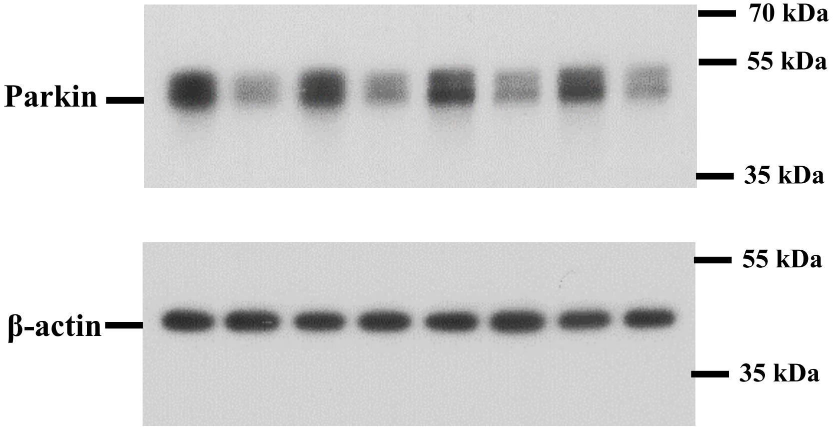

	WHITE				BEIGE			
siCTRL	+	-	+	-	+	-	+	-
siPARK2	-	+	-	+	-	+	-	+
cAMP	-	-	+	+	-	-	+	+

Primary

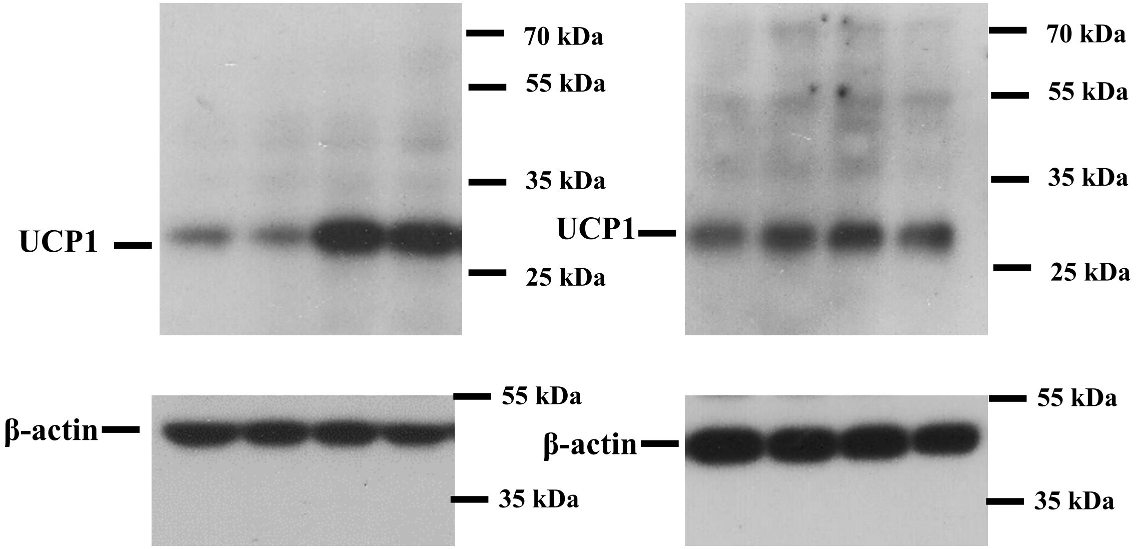

	WHITE			
siCTRL	+	-	+	-
siPARK2	-	+	-	+
cAMP	-	-	+	+

	BEIGE			
siCTRL	+	-	+	-
siPARK2	-	+	-	+
cAMP	-	-	+	+

Original western blots for Figure 6

Primary

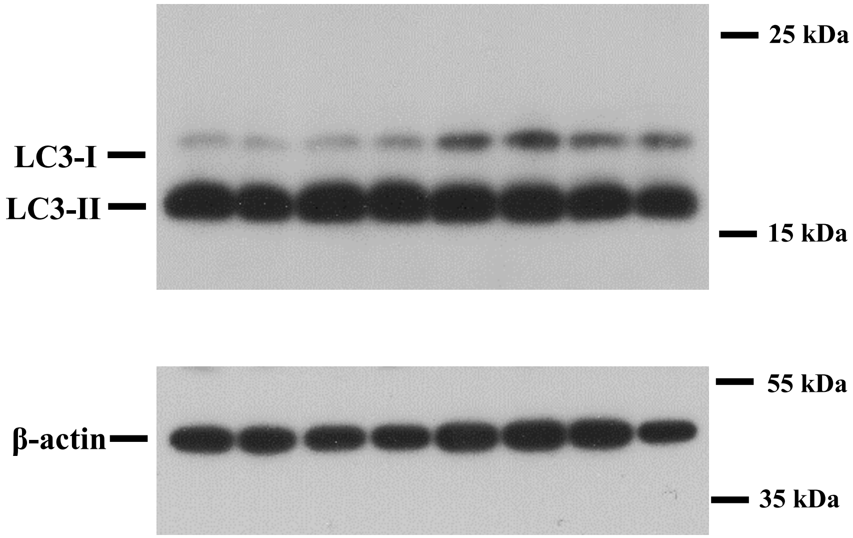

	WHITE				BEIGE			
siCTRL	+	-	+	-	+	-	+	-
siPARK2	-	+	-	+	-	+	-	+
cAMP	-	-	+	+	-	-	+	+

Primary

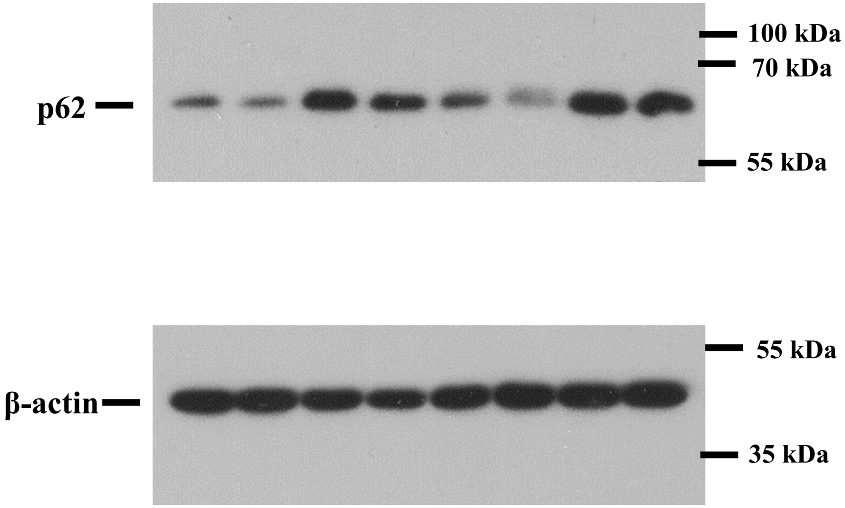

	WHITE				BEIGE			
siCTRL	+	-	+	-	+	-	+	-
siPARK2	-	+	-	+	-	+	-	+
cAMP	-	-	+	+	-	-	+	+

Original western blots for Figure 6

Primary

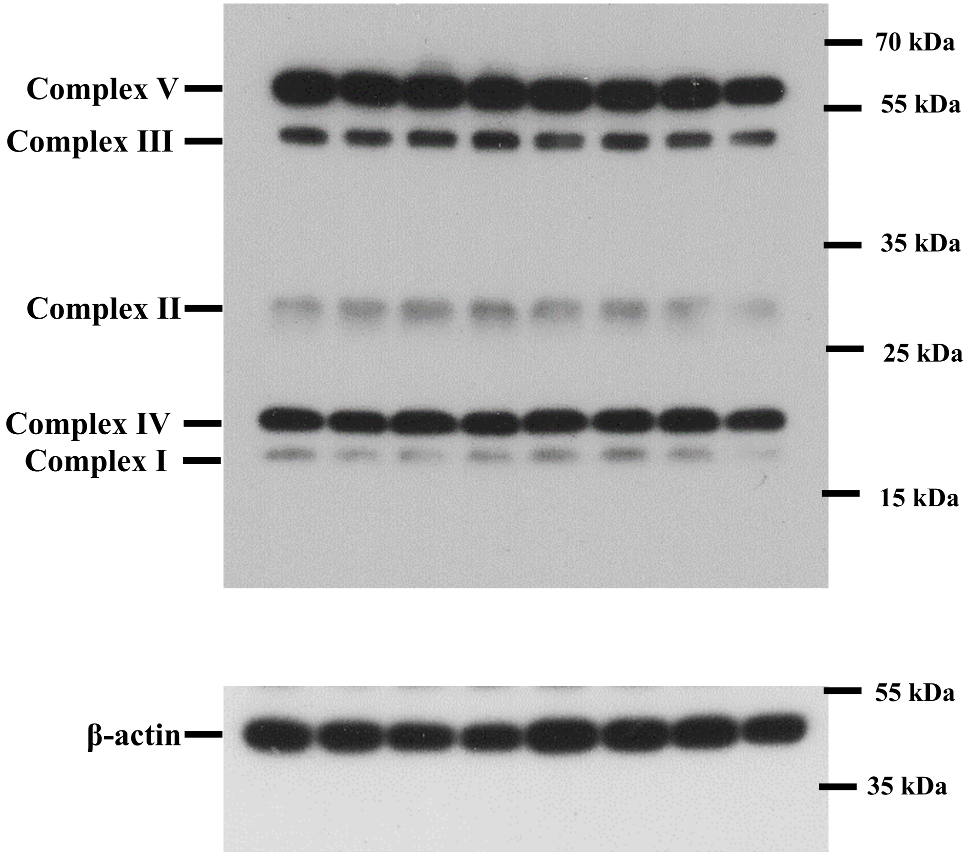

	WHITE				BEIGE			
siCTRL	+	-	+	-	+	-	+	-
siPARK2	-	+	-	+	-	+	-	+
cAMP	-	-	+	+	-	-	+	+
